# Treatment Outcomes of Patients with Orbital Inflammatory Diseases: Should Steroids Still Be the First Choice?

**DOI:** 10.3390/jcm13143998

**Published:** 2024-07-09

**Authors:** Karim Al-Ghazzawi, Inga Neumann, Mareile Knetsch, Ying Chen, Benjamin Wilde, Nikolaos E. Bechrakis, Anja Eckstein, Michael Oeverhaus

**Affiliations:** 1 Department of Ophthalmology, University Hospital Essen, 45147 Essen, Germany; 2 Department of Nephrology, University Hospital Essen, 45147 Essen, Germany

**Keywords:** NSOI, IgG4-ROD, OID, inflammation, biologicals, steroids, myositis, dacryoadenitis, lacrimal gland

## Abstract

**Objective**: To clarify the therapy response in orbital inflammatory diseases (OID), we analyzed the treatment effects of steroid therapy, the use of disease-modifying antirheumatic drugs (DMARDS), and biologicals in our tertiary referral center cohort. **Methods**: We collected the clinical and demographic data of all patients treated for non-specific orbital inflammation (NSOI) (*n* = 111) and IgG4-ROD (*n* = 13), respectively at our center from 2008 to 2020 and analyzed them with descriptive statistics. NSOI were sub-grouped according to the location into either idiopathic dacryoadenitis (DAs) (*n* = 78) or typical idiopathic orbital myositis (*n* = 32). **Results**: Mean age at first clinical manifestation was significantly different between subgroups (IOI: 49.5 ± 18, IgG4-ROD: 63.2 ± 14, *p* = 0.0171). Among all examined OID, 63 patients (50%) achieved full remission (FR) with corticosteroids (NSOI 53%/IgG4-ROD 31%). In contrast, classic myositis showed a significantly higher response (76%). Disease-modifying drugs (DMARDS) for myositis accomplished only 33% FR (NSOI 57%) and 66% did not respond sufficiently (NSOI 43%). The biologic agent (Rituximab) was significantly more efficient: 19 of 23 patients (82%) achieved full remission and only 4 (17%) did not respond fully and needed orbital irradiation or orbital decompressive surgery.

## 1. Introduction

Orbital inflammatory diseases (OID) encompass a wide range of pathologies, including isolated diseases such as IgG4-related orbital disease (IgG4-ROD), non-specific orbital inflammation (NSOI, formerly orbital pseudotumor), and manifestations of systemic diseases such as the most common thyroid eye disease (TED), granulomatosis with polyangiitis (GPA), Sjögren syndrome (SjS), and sarcoidosis [1,2,3,4], among others. They can affect any tissue of the orbit focally or diffusely, with presentations ranging from abrupt to insidious onset. Symptoms vary depending on the affected tissues, but typically include pain, proptosis, periorbital edema and erythema, impaired motility, and consequently diplopia in most cases, and sometimes decreased visual acuity [1,3,5]. Differential diagnosis includes isolated and systemic autoimmune diseases, lymphoproliferative diseases, and infectious diseases [6,7]. Therefore, an extensive laboratory and imaging workup, including autoimmune markers, is recommended [1,8,9]. Some specific autoimmune conditions can be identified in this way (TED, GPA, classical ocular myositis). However, in all other cases, diagnosis relies mostly on histopathological findings after orbital biopsy [10]. In the future, advances in MRI technology and AI-based diagnostics might overcome this hurdle [11]. Despite all advances, NSOI remains an exclusion diagnosis [1]. Classical myositis is usually treated with corticosteroids without performing a biopsy first [1,12]. Dose regimens vary due to the lack of an international or European guideline (20–80 mg/day or 1 mg/kg body weight per day, tapered) [9]. Typical myositis subsides under treatment within 2–3 days.

In contrast, treatment of NSOI and IgG4-ROD is challenging. Some patients respond very well to (a) systemic steroid therapy, while others need multiple cycles and (b) additional immunosuppressive agents (DMARDs) as maintenance therapy to prevent relapses and achieve a stable state; however, if still not responsive to therapy (c) biologicals can be applied usually in cases with recalcitrant nonspecific orbital inflammation. Some even require (d) irradiation or debulking surgery [1,13]. Patients might be spared unnecessary recurrences if treated early with effective immunosuppressive treatments, but currently, predictive factors are still missing [14,15,16]. Immunosuppressive Agents (DMARDs) are beneficial for patients with non-responsiveness or recurrence post-corticosteroid therapy: Methotrexate; Cyclosporin-A; Mycophenolate mofetil (MMF); Cyclophosphamide, Sulfasalazine, and Azathioprine (AZA) [17,18]. Since IgG4-ROD shows a high relapse rate of about 50% [19,20], MTX, AZA, MMF, Infliximab, Cyclophosphamide, and Rituximab (RTX) should be considered early for effective treatment [21]. Biologicals such as RTX seem to be most effective for IgG4-ROD, with up to 94% remission in a recent review [22]. Comparable results have also been achieved in NSOI patients [23]. Alternatively, MTX, MMF, tocilizumab, infliximab, and adalimumab can be used for NSOI [1,24]. Unfortunately, only a few small randomized controlled trials are available for NSOI and IgG4-ROD. Thus, most treatments are ‘off-label’ and financial coverage needs to be applied for with health insurance companies. Only GPA patients benefit from the approval of the EMA for RTX in 2013, and long-term data are available [25]. Therefore, we aimed to analyze our tertiary referral center cohort of patients with NSOI and IgG4-ROD for treatment effects in terms of stable disease and possible clinical predictors for the effectiveness of the different therapeutic modalities.

## 2. Patients and Methods

### 2.1. Study Population

We identified 127 patients with typical clinical course and certain diagnoses for NSOI (*n* = 114), and IgG4-ROD (*n* = 13) from our patient database comprised of patient records between 2008 and 2020. NSOI were sub-grouped into either typical idiopathic dacryoadenitis (idiopathic DAs) (*n* = 78) or idiopathic orbital myositis (*n* = 32). The study was performed under adherence to the ethical foundations of the Declaration of Helsinki and was approved by the Ethics Commission of the University of Essen (11-4822-B0). Diagnosis of NSOI and IgG4-ROD were based on clinical, flow cytometric, and histological (including immunostaining) examinations. IgG4-ROD was diagnosed in accordance with the published 2020 revised comprehensive diagnostic (RCD) criteria [26]. Briefly, IgG4-ROD was diagnosed in the presence of (1) one or more organs showing diffuse or localized swelling or a mass or nodule characteristic of IgG4-RD. In single-organ involvement, lymph node swelling is omitted. (2) Serum IgG4 levels greater than 135 mg/dL. (3) Positivity for two of the following three criteria: (a) dense lymphocyte and plasma cell infiltration with fibrosis; (b) ratio of IgG4-positive plasma cells/IgG-positive cells greater than 40% and the number of IgG4-positive plasma cells greater than 10 per high-powered field; and (c) typical tissue fibrosis, particularly storiform fibrosis, or obliterative phlebitis. 

Patients who fulfilled all 3 criteria were considered as definitive IgG4-RD. Patients with (1) and (2) or (1) and (3) were regarded as definitive IgG4-RD if they fulfill the organ-specific criteria for IgG4-RD. Cases that did not meet the inclusion criteria or had incomplete datasets (loss to follow-up) were excluded.

### 2.2. Statistical Evaluation

To analyze metric data, median values (x^~^) and range or mean and standard deviation (SD) were computed. A student’s *t*-test (two-tailed) was used to assess differences between groups if the D’Agostino–Pearson omnibus normality test indicated normal distribution; otherwise, the Mann–Whitney Test was used. Fisher’s exact test was used to examine group distributions of binary variables. For the comparison of ordinal variables and factors with more than two groups, either the Kruskal–Wallis test (non-parametric) or ANOVA (parametric) were used to detect group differences. All calculations were performed with SPSS (IBM SPSS Statistics, Chicago, IL, USA, Version 22.0.0,) and Graph Pad Prism (Prism 9 for Windows, Software Inc., San Diego, CA, USA, Version 9.0.0). *p*-values are given descriptively without α-adjustment for multiple testing. 

### 2.3. Clinical Data Collection

General information was collected from the medical records database at baseline and follow-up examinations, including age, gender, affected eye, symptoms, previous history (including previous glucocorticoid therapy prior to referral), clinical manifestations, serum blood results, imaging findings, immunohistochemical indicators, and given treatments. Pathological diagnosis was confirmed by a minimum of two pathologists. If histopathological diagnosis was uncertain a tertiary referral pathologist of an independent center was consulted. 

### 2.4. Imaging and Biopsy

Prior to biopsy, all patients received an orbital imaging modality. Magnetic resonance imaging (MRI) and/or computed tomography (CT), were obtained and evaluated by neuroradiologists. A biopsy of the lesions was performed when it was considered necessary to confirm the diagnosis. If extra-ophthalmic manifestation was suspected, systemic imaging was performed.

### 2.5. Treatment Protocol

#### 2.5.1. Glucocorticosteroids (GC)

The GC-tapering regimen varied. Depending on the severity of the NSOI ranging GC pulse was started with a dose between 0.75 mg prednisolone/Kg Bodyweight and 1.5 mg prednisolone/Kg Bodyweight and tapered off slowly over weeks. 

#### 2.5.2. DMARDs

Therapy was only commenced after ruling out contraindications e.g., lymphopenia, systemic severe infections, latent tuberculosis (TBC), uncontrolled cardiac disease, and pregnancy. In addition, the vaccination status was optimized.

##### Mycophenolate Mofetil

Mycophenolate Mofetil was given at a dose of 2 × 360 mg per day, orally. The recommendation was given for separate doses per day taken with meals to improve gastrointestinal tolerance. Mycophenolate leads to a relatively selective inhibition of DNA replication in T- and B-cells. 

##### Methotrexate

Methotrexate was initiated at the same time as Corticosteroids at a dose of 15–20 mg per week, orally or preferably subcutaneously, along with folic acid supplementation. The drug inhibits dihydrofolate reductase and suppresses both B- and T-cells.

##### Cyclophosphamide

Cyclophosphamide was given at a dose of 15 mg/kg as pulse therapy over two to four cycles or as an oral continuous therapy at 2 mg/kg/d. Its cytotoxic effect is mainly due to cross-linking of DNA strands (alkylating agent), therefore inhibiting protein synthesis. 

##### Cyclosporin A

Cyclosporin A was given was given to patients at a starting dose of 4 mg/kg/day and tapered to 2 mg/kg/day. The drug suppresses lymphocyte-mediated responses, inhibits T cells, and decreases the production of IL-1 and IL-2.

##### Azathioprine

Azathioprine was given was given to patients in a dose of 2–3 mg/kg/day. It is an antagonist of purines, resulting in the inhibition of DNA, RNA, and protein synthesis.

##### Sulfasalazine

Sulfasalazine exerts its anti-inflammatory effects through multiple mechanisms. Proposed mechanisms of action: inhibition of the transcription factor nuclear factor kappa-B (NF-kB), which leads to the suppression of NF-kB responsive pro-inflammatory genes, including TNF-α. Additionally, sulfasalazine induces caspase 8-induced apoptosis in macrophages, thereby inhibiting TNF-α expression. 

#### 2.5.3. Biologicals

##### Rituximab (CD-20 Inhibitor)

RTX is a monoclonal antibody that targets the CD20 antigen found on the surface of B cells. The working principle of rituximab involves: 1. Depletion of B Cells: Rituximab binds to the CD20 antigen on the surface of B cells, leading to the destruction of these cells. 2. Modulation of Immune Response: By targeting B cells, rituximab can modulate the immune response. 3. Impact on Autoimmune Diseases: In autoimmune diseases, B-cells play a role in the production of autoantibodies and the presentation of autoantigens. Rituximab’s ability to deplete B cells can help in reducing autoantibody levels and suppressing the autoimmune response. It was given as an off-label treatment to patients. Rituximab was given as 2 intravenous doses of (500 mg to 1 g) 2 weeks apart in addition to standard treatment. 

##### Treatment Outcome

We defined therapy response to corticosteroids as follows:

- Full Remission: Patients who responded to corticosteroids over a maximum of two courses with a remission of clinical symptoms and pain without a recurrence over the observed time of this study;
- Partial Remission: Patients that responded initially to corticosteroids with a remission of clinical symptoms, but needed a second immunosuppressive agent (DMARDs or Biologicals) due to incomplete response recurrence dosage >10 mg.

We defined therapy response to steroids in combination with DMARDS as follows:

- Full Remission: Patients that responded to a combination of corticosteroids (maximum 7.5 mg/day) with DMARDS, without a recurrence over the observed time of this study;
- Partial Remission: Patients that responded initially to a combination of higher-dosed corticosteroids with DMARDS with a remission of clinical symptoms but needed treatment with Biologicals due to incomplete response or recurrence of disease with steroids >10 mg.

We defined therapy response to biologicals as follows:

- Full Remission: Patients that responded to a combination of corticosteroids (maximum 7.5 mg/day) with biologicals without a recurrence over the observed time of this study;
- Partial Remission: Patients that did not respond sufficiently to treatment with biologicals and corticosteroids (>7.5 mg/day).

## 3. Results

### 3.1. Study Population and Characteristics of OID

The 124 patients ranged in age from 9 to 91 years (mean 51.9 years); 55 were male, and 69 were female. Observed age as well as sex predilection was different between observed entities, *p* = 0.04 and *p* = 0.03, respectively (Table 1). The left eye was affected in 55 patients, the right eye in 58 patients, and both eyes in 11 patients. In 78 out of 127 (62%) cases, a biopsy was performed in our institution to confirm the diagnosis in addition to clinical presentation. Forty cases were treated without a biopsy. The remaining 14 patients had external biopsies, a clear diagnosis due to a diagnosed systemic disease, or refused to undergo surgery due to comorbidities. Idiopathic DAs were more likely to be biopsied *p* < 0.0001 (Table 1).

The median duration of time between first clinical presentation and biopsy was 6.9 ± 5 months. The patients presented with a range of signs and symptoms. A swelling/mass was the most common presentation other than proptosis, pain, extraocular muscle restriction, diplopia, ptosis, and decreased vision (Table 2).

### 3.2. Therapy Response

#### 3.2.1. Classical Myositis

**Figure 1:**
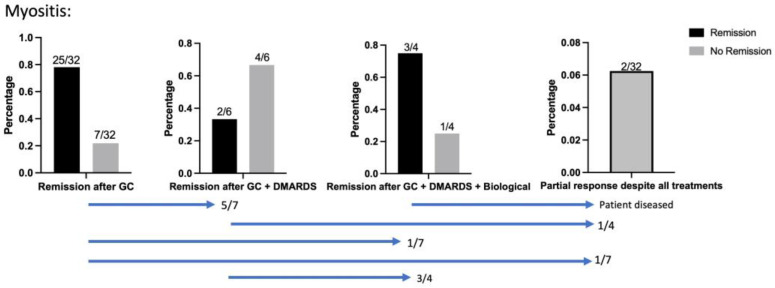
Treatment efficacy in Myositis cases. Blue bars show patient flow between cohorts; 25/32 achieved remission after a maximum of two corticosteroid courses, 8/25 (32%) being male patients. Five patients were subjected to GC + DMARDs, of whom two patients achieved remission. Four patients treated with GC + DMARDs needed treatment with RTX to achieve remission.

Response to corticosteroids was very high in classical myositis when compared to the overall study population (Figure 1): Most patients (78%) achieved remission after a maximum of two corticosteroid courses (Table 3). Two patients achieved remission after GC + DMARDS, but four patients needed treatment with RTX. All four unresponsive cases to DMARDs were biopsied to rule out malignancies before therapy with RTX. Despite all treatments, 2/32 patients only responded partially and were therefore subjected to radiation therapy. One patient who responded initially while being treated with RTX, but who diseased before the remission of clinical symptoms could be achieved, was counted as partial remission. 

#### 3.2.2. Treatment Efficacy in Dacryoadenitis

**Figure 2:**
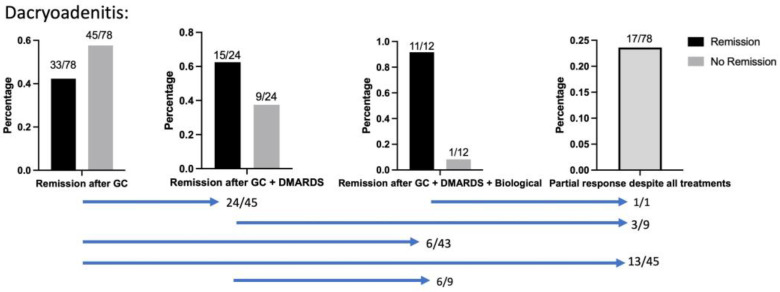
Definitive therapy in the examined DAs. GC achieved remission in 42%, and DMARDS (Cyclophosphamid, Azathioprin, MTX, or Cyclosporin A) achieved remission in 62.5%. Remission was achieved with Rituximab in 91%. Blue bars show patient flow between cohorts; Despite all treatments, 19/78 patients could not achieve remission (24%). A biopsy to rule out malignancies (e.g., Lymphoma) was performed in all patients treated with DMARDs, Biologicals, as well as nonresponsive recalcitrant DAs. In total, 64/79 DAs were biopsied.

After treatment with corticosteroids, inactive disease was achieved in 42% of 78 patients with DA (Figure 2). GC + DMARDS (Cyclophosphamides, Azathioprine, MTX, and Cyclosporin A) were applied in 24/78 cases. Of these, 63% of patients achieved inactive disease. MTX (15 mg/week subcutaneous; in single cases 20 mg/week for 4–40 months) was used as monotherapy in 14/24 cases. Both Cyclophosphamide (1 g/4-courses) and Mycophenolate monotherapy (350–750 mg/day for 6–36 months) were noted in 2/24 cases (1 g over 2–3 courses/(750 mg/day)). Azathioprine (150 mg–200 mg/day for 4 months) and Cyclosporin (4–2 mg/kg bodyweight/day for 2 months) were used in one case, respectively. The therapy regimen MTX + Azathioprine (15–20 mg/weekly + 150–200 mg/day for 4–40 months), MTX + MMF (20 mg/day + 500 mg/day for 24 months), or MTX + Sulfasalazine (20 mg/day + 500 mg/day for 36 months) was noted in 6/24 cases. Insufficient response to DMARDs and therapeutic switch to a biological (Rituximab) was necessary in 6/24 cases. Complete therapeutic switch to biologicals (Rituximab) due to insufficient response to Corticosteroids while organ-threatening orbital disease was observed was necessary in 7.6% (6/78) of cases. Biologicals (Rituximab) were applied in 12 DA patients. Here, full remission was achieved in 91% (11/12), of which one patient showed only partial response despite all treatments. Altogether 17/78 (24%) patients responded only partially despite all treatments. Here, (10/17) were treated with radiation therapy of which 7 received additional surgical decompression. In all 14/17 patients were surgically decompressed due to organ-threatening behavior of the orbital mass. Supplemental Figure S1 demonstrates the time of relapse under DMARD vs. GC therapy. In patients with recurrent DAs, the interval between the Inflammatory episode and the recurrence under therapy was shorter in GC (86% relapse-free survival in DMARDs vs. 64% relapse-free survival in GC after 2 months of treatment).

#### 3.2.3. IgG4-ROD

**Figure 3:**
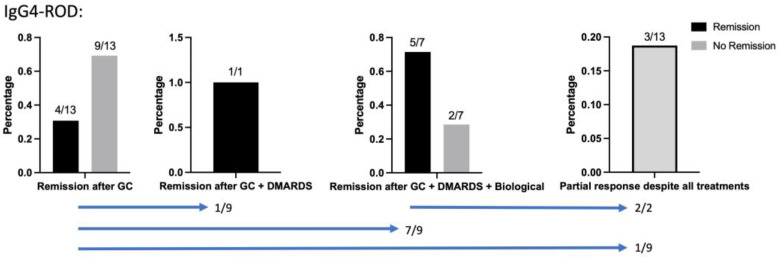
Response to all therapies in examined IgG4-ROD cases. Blue bars show patient flow between cohorts; 9/13 patients relapsed when corticosteroids were tapered and were therefore subjected to other therapies. One relapsed patient received DMARDS monotherapy, which achieved remission. Rituximab was given to the other seven relapsed patients and achieved remission in 5/7 cases; 3/13 patients were subjected to decompressive surgery in organ-threatening disease manifestations.

The initial response to corticosteroids was 100%. However, 69% of patients relapsed when corticosteroids were tapered (prednisolone 7.5 mg, Figure 3). One relapsed patient received DMARDS (Mycophenolate 750 mg for 3 years) monotherapy, which achieved remission. Rituximab was given to the other seven relapsed patients and achieved remission in 71%. Decompressive surgery was necessary in three patients due to insufficient response despite all treatments.

## 4. Discussion

The results of this retrospective analysis of patient data from 124 patients with NSOI (DA and Myositis) and IgG4 from our tertiary referral center showed significantly different treatment outcomes, depending on the disease entity and localization. Whereas orbital myositis mostly showed promising remission with GC monotherapy, idiopathic dacryoadenitis showed a much higher relapse rate and demanded second-line treatments, such as DMARDs and biologicals. Unlike other organ manifestations, which seem to respond well to GC monotherapy, our small group of isolated IgG4-ROD needed second-line therapies in two-thirds of the patients and even surgery to avoid dramatic consequences for the visual function.

### 4.1. Treatment of NSOI

First-line treatment for NSOI remains high-dose corticosteroids, tapered off slowly over months. Often the tapering is carried out too quickly, resulting in a “relapse”, which is more an improper treatment of the orbital inflammation. GCs come with the perks of being affordable, easily accessible, and quite effective in addressing NSOIs. In patients that show positive responses to the drug, complaints, and especially pain start to diminish quickly.

#### 4.1.1. Myositis

There are no well-designed, randomized controlled clinical trials for the treatment of myositis, and most publications are small, retrospective case studies. NSAIDs have been used in the past, though GCs are still first-line therapy, and currently colleagues have reported increased use of DMARDs (Supplemental Table S1 includes several publications with myositis cases). GCs were reported to be effective in 60–74% of cases. DMARDs (Azathioprine and MTX) have been reported effective in 60–80%, but have only been administered in a few cases (seven cases in total). Biologicals have been reported to be effective in irresponsive cases with multiple relapses. This was also true in our study cohort, which points out the positive effect of Biologicals (RTX) in relapsing myositis cases. In addition, the biological TNF-alpha (Infliximab) has been reported effective in chronic orbital myositis [27] with promising results (71% remission). However, due to small patient numbers, further studies of this agent for recurring orbital myositis cases are needed. To the best of our knowledge, our study is the first study with the largest patient cohort illustrating detailed therapy responses to each medication.

#### 4.1.2. Dacryoadenitis

The observed response rate was quite low in GC monotherapy with only 42%. This is in accordance with previous literature reporting an estimate of 20% to 60% recurrence in patients with dacryoadenitis (Supplemental Table S2) [10,28,29]. Due to this low remission rate for idiopathic DAs, an additional immunosuppressive agent (DMARD) is recommended and therapeutic preparation (e.g., blood examination to rule out contraindications) should be performed very early [24,30]. The side-effect profile of long-term corticosteroids, includes adrenal insufficiency, cataract, osteoporosis, psychosis, diabetes, and gastrointestinal disorders and should not be underestimated [31]. Typical DMARD medication includes cyclosporine-A, methotrexate, cyclophosphamide, tacrolimus, azathioprine, and mycophenolate mofetil [32,33,34]. The patient number per specific DMARD was rather low, which is why further studies are needed to elucidate the response to the monotherapies. Methotrexate is the most used steroid-sparing immunomodulating agent for the management of orbital inflammation, most probably due to its low risk/benefit ratio. Side effects include fatigue, hair loss, gastrointestinal disturbance, and elevated liver enzymes [35]. Supplementing dietary folate and regular monitoring of liver enzymes are needed to minimize these adverse effects is recommended.

Monoclonal antibodies have now been highlighted as novel immunomodulating agents. These biologic drugs are highly specific and have proven to be superior to conventional immunosuppressive drugs regarding their efficacy and safety for specific indications in more common autoimmune diseases. We analyzed 12 cases with Rituximab and could show its effectiveness as a second-line treatment (90% remission). This is in line with previously published results [14,23]. The excellent response compared to other DMARDs should lead to an early consideration of RTX. Recurring autoantibodies in patients who present with NSOI, in combination with its association with immunological inflammatory disorders, e.g., Crohn’s disease, rheumatoid arthritis, diabetes mellitus, systemic lupus erythematosus, hints at an underlying autoimmune process [36]. Some authors even suggest RTX as first-line therapy in idiopathic DAs, since patients often show complete remission after one cycle, preventing fibrosis in adipose tissue and complicated courses [16,23]. TNF-alpha blockers have been reported effective in the treatment of irresponsive, relapsing NSOI [37]. Our study also points out the efficacy of success over time when observing relapse-free survival (Supplemental Figure S1). Altogether, both therapy success and observed relapse-free survival are proving a more efficient therapy in DMARDs and biologicals compared to GC monotherapy. Relapsing drug-resistant cases were observed in 17 cases for our examined DA cohort. For these patients, only radiation and surgery remain, especially in sight-threatening manifestations. Radiation has historically been considered an effective alternative in recalcitrant or recurrent NSOIs [38]. Surgery has been proposed to be effective in infiltrative fibrosing non-responsive NSOIs [39]. Further studies are needed to affirm the theory that some patients with NSOIs might profit from early therapy with biologicals, especially before fibrosis within the adipose tissue occurs. Improved orbital imaging and molecular profiling of biopsies might be crucial to determine which patients can profit from early biologicals [7]. 

### 4.2. Treatment of IgG4-ROD

Patients with IgG4-related disease (IgG-RD) typically respond well at first to corticosteroids, especially in the early phases of the disease [40]. Serum IgG4 levels, lymphocytic infiltration, as well as clinical symptoms such as organ enlargement, pain, or diplopia usually improve within the first weeks. In an international consensus, GC is therefore recommended as a first-line induction treatment [41]. A small minority of relapsing patients, however, do need a second immunosuppressive agent [42]. In patients with orbital manifestation, this seems to be different at least in our cohort. In our cohort, 38% of patients showed remission with only GC. However, the majority (62%) relapsed when corticosteroids were tapered. This is why it is recommended to consider DMARDs early on in IgG4-ROD similar to idiopathic DAs [21]. The relapsed patients of our cohort were either treated with DMARDs, debulking surgery, or Rituximab. The last achieved remission in 71% of cases, compared to only 25% in DMARDs. This is a bit lower compared to other studies analyzing the effects of RTX (94%) but could be rather explained by the small number of patients [22]. All observed patients who achieved remission showed a stable remission after one or two courses of RTX without late recurrences that demanded additional immunosuppressive agents. This might indicate that IgG4-ROD patients should be evaluated early on in case of a relapse after GCs for RTX treatment to avoid a chronic treatment with DMARDs. Further studies are needed to confirm the observed long-term effect of RTX on IgG4-ROD after 1–2 courses. 

### 4.3. Limitations

The interpretation of our results is limited by its retrospective design and the typical. However, due to the rather high amount of patients considering the low incidence of the diseases, we think that our data are very useful to improve OID therapy. Future studies should be planned in a multicentric prospective design to further elucidate this matter. Ideally, they should include elaborate imaging protocols and molecular testing of the diseased patients. 

## 5. Conclusions

Our single-center retrospective analysis emphasized the difficulty of treating patients with OIDs. Corticosteroids were confirmed as a viable option for idiopathic orbital myositis and induction therapy for patients with more severe NSOI and IgG4-ROD; however, the common relapses in these patients demonstrate the need for an early alternative immunosuppressive therapy. DMARDs are shown to be only partially effective. RTX, on the other hand, was, in our cohorts, the most effective second-line treatment and should be considered as an early second-line option, especially due to the excellent long-term results.

## Figures and Tables

**Table 1:** Population characteristics in our index population stratified for each disease entity and subtype of disease.

	All OIDs	Myositis	Idiopathic DA *n* = 79	IgG4-ROD *n* = 13	*p*
Age	51.9 ± 17.76	49.26 ± 16.08	49.67 ± 18.7	63 ± 13.83	0.0492 ^a^
Unilateral manifestation	113 (91.2%)	28	72	13	0.42 ^b^
Male sex	55 (44.3%)	12	38	5	0.0392 ^b^
Biopsy	84 (67.74%)	7	64	13	0.0001 ^b^

Unless otherwise stated, data are means ± SD or are proportions (%) or counts ^a^: Welch ANOVA test. ^b^: Kruskal–Wallis test.

**Table 2:** Clinical symptoms present in our index population stratified for each disease entity and subtype of disease.

Entity	Eyelid Swelling	Proptosis	Limited Eye Movement	Visual Loss	Diplopia	Orbital Pain
Myositis *n* = 32	23 (72%)	8(25%)	15 (47%)	8 (25%)	13 (41%)	26 (81%)
idiopathic DAs *n* = 79	53(67%)	53(67%)	44 (56%)	31 (39%)	50 (63%)	64 (81%)
IgG4-ROD *n* = 13	8(62%)	8(62)	7 (54%)	8 (62%)	5 (38%)	6 (46%)

**Table 3:** Clinical remission after treatment in our index population stratified for each disease entity and subtype of treatment. Stated data are proportions (%). ^a^: Fisher’s exact test in glucocorticoids vs. group.

	All	Remission after Treatment	No Remission	*p*
Myositis	32	30	2	
Glucocorticoids	32	(78%) 25	(22%) 7	
DMARDs	6	(33.3%) 2	(66.7%) 4	0.046 ^a^
Biologicals	4	(75%) 3	(25%) 1	1 ^a^
Idiopathic DAs	78	61	17	
Glucocorticoids	78	(42%) 33	(58%) 43	
DMARDs	24	(62.5%) 15	(37.5%) 9	0.3571 ^a^
Biologicals	12	(92%) 11	(8%) 1	0.0035 ^a^
IgG4-ROD	13	10	3	
Glucocorticoids	13	(30%) 4	(70%) 9	
DMARDs	1	(100%)1	0	0.357 ^a^
Biologicals	7	(71%) 5	(19%) 2	0.15 ^a^

## Data Availability

The data that support the findings of this study are available from the corresponding author, M.O., upon reasonable request.

## Supplementary Materials

The following supporting information can be downloaded at: https://www.mdpi.com/article/10.3390/jcm13143998/s1. Figure S1. Proportions of relapse free survival: CS vs. DMARDs in DAs. Table S1. Literature Summary of Patients reported with Orbital Myositis and treatment outcomes. Table S2. Literature Summary of Patients reported with isolated Dacryoadenitis and treatment outcomes. References [43,44,45,46,47,48,49] are cited in the Supplementary Materials.
