# Supplementary material for: Treatment Outcomes of Patients with Orbital Inflammatory Diseases: Should Steroids Still Be the First Choice?
Source: J Clin Med. 2024 Jul 9;13(14):3998. doi: 10.3390/jcm13143998 (PMC11277562; doi:10.3390/jcm13143998)
Supplement: Supplementary file 1 [file jcm-13-03998-s001.zip › jcm-3022508-supplementary.pdf]

Journal of
Clinical Medicine

MDPI

Supplementary Materials

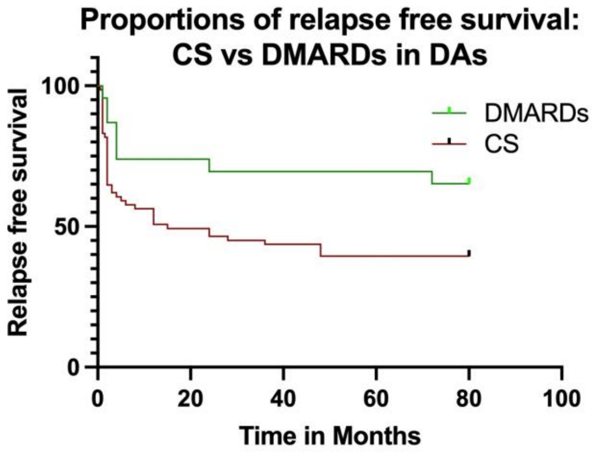

Figure S1. Proportions of relapse free survival: CS vs. DMARDs in DAs.

Table S1. Literature Summary of Patients reported with Orbital Myositis and treatment outcomes.

Study/Author	Patients	Drug	Response Rates
Geva E. Mannor et al. [1]	26	NSAIDs	85%
		GC	60%
		RT	50%
Halimi et al. [2]	10	GC<br>NSAIDs	60%
I Mombaerts et al. [3]	16	GC, NSAIDs & RT	54%
F Montagnese et al. [4]	7	GC<br>Azathioprin&MTX	60%<br>60%
Min Seok Kang et al. [5]	31	GC<br>Azathioprin&MTX(5)<br>Biologicals(1)	74%<br>NA<br>NA
Garrity et al. [6]	7	Infliximab	71%

GC = oral corticosteroids; NSAID = nonsteroidal anti-inflammatory drugs; MTX = Methotrexate; RT = radiotherapy. NA: Not available.

Table S2. Literature Summary of Patients reported with isolated Dacryoadenitis and treatment outcomes.

Study/Author	Patients	Drug	Response Rates
H. Andrew et al. [7]	79	NSAIDs (1)<br>GC (55)<br>MTX (5) & Azathioprin (3)	63% (cumulative)
Kubota T. et al. [8]	45 (DAs)	GC	53–74%
Yuen et al. [9]	21 (DAs)	NA.	NA.
Swamy BN et al. [10]	24 (NSOIs)	GC (24)<br>GC & DMARD (7)	66% (cumulative)
Lee, Min Joung et al. [11]	72 (NSOIs)	GC (44)<br>GC & MTX (7)<br>MTX (1)	66.7% (cumulative)

GC = oral corticosteroids; NSAID = nonsteroidal anti-inflammatory drugs; MTX = Methotrexate. NA: Not available.

References

1. Mannor, G.E.; Rose, G.E.; Moseley, I.F.; Wright,J.E. Outcome of orbital myositis: clinical features associated with recurrence. *Ophthalmology* **1997**, *104*, 409–414.
2. Halimi, E.; Rosenberg, R.; Wavreille, O.; Bouckehove, S.; Franquet, N.; Labalette, P. Présentation clinique et prise en charge des myosites aiguës dans les inflammations orbitaires non spécifiques [Clinical features and management of acute myositis in idiopathic orbital inflammation]. *J. Fr. Ophtalmol.* 2013, 36, 567–574. https://doi.org/10.1016/j.jfo.2012.09.012. (in French)
3. Mombaerts, I.; Schlingemann, R.O.; Goldschmeding, R.; Koornneef, L. Are systemic corticosteroids useful in the management of orbital pseudotumors? *Ophthalmology* **1996**, *103*, 521–528. https://doi.org/10.1016/s0161-6420(96)30663-5.
4. Montagnese, F.; Wenninger, S.; Schoser, B. Orbiting around" the orbital myositis: clinical features, differential diagnosis and therapy. *J. Neurol.* **2016**, *263*, 631–640. https://doi.org/10.1007/s00415-015-7926-x.
5. Kang, M.S.; Yang, H.K.; Kim, N.; Hwang, J.-M. Clinical Features of Ocular Motility in Idiopathic Orbital Myositis. *J. Clin. Med.* **2020**, *9*, 1165. https://doi.org/10.3390/jcm9041165.
6. Garrity, J.A.; Coleman, A.W.; Matteson, E.L.; Eggenberger, E.R.; Waitzman, D.M. Treatment of recalcitrant idiopathic orbital inflammation (chronic orbital myositis) with infliximab. *Am. J. Ophthalmol.* **2004**, *138*, 925–930. https://doi.org/10.1016/j.ajo.2004.06.077.
7. Andrew, N.H.; Kearney, D.; Sladden, N.; McKelvie, P.; Wu, A.; Sun, M.T.; McNab, A.; Selva, D. Idiopathic Dacryoadenitis: Clinical Features, Histopathology, and Treatment Outcomes. *Am. Jounral Ophthalmol.* **2016**, *163*, 148–153.E1. https://doi.org/10.1016/j.ajo.2015.11.032.
8. Kubota, T.; Iwakoshi, A. Clinical heterogeneity between two subgroups of patients with idiopathic orbital inflammation. *BMJ Open Ophthalmol.* **2022**, *7*, e001005. https://doi.org/10.1136/bmjophth-2022-001005.
9. Yuen, S.J.A.; Rubin, P.A.D. Idiopathic Orbital Inflammation: Distribution, Clinical Features, and Treatment Outcome. *Arch. Ophthalmol.* **2003**, *121*, 491–499. https://doi.org/10.1001/archopht.121.4.491.
10. Swamy, B.N.; McCluskey, P.; *a*Nemet, A.; Crouch, R.; Martin, P.; Benger, R.; Ghabriel, R.; Wakefield, D. Idiopathic orbital inflammatory syndrome: Clinical features and treatment outcomes. *Br. J. Ophthalmol.* **2007**, *91*, 1667–1670. https://doi.org/10.1136/bjo.2007.124156.
11. Lee, M.J.; Planck, S.R.; Choi, D.; Harrington, C.A.; Wilson, D.J.; Dailey, R.A.; Ng, J.D.; Steele, E.A.; Hamilton, B.E.; Khwarg, S.I.; et al. Non-specific orbital inflammation: Current understanding and unmet needs. *Prog. Retin. Eye Res.* **2021**, *81*, 100885. https://doi.org/10.1016/j.preteyeres.2020.100885.
